# The effects of mouthwashes in human gingiva epithelial progenitor (HGEPp) cells

**DOI:** 10.1007/s00784-022-04422-z

**Published:** 2022-03-07

**Authors:** Zsófia Kőhidai, Angéla Takács, Eszter Lajkó, Zoltán Géczi, Éva Pállinger, Orsolya Láng, László Kőhidai

**Affiliations:** 1 Department of Genetics, Cell- and Immunobiology, Semmelweis University, Budapest, Hungary; 2 Department of Oral Diagnostics, Semmelweis University, Budapest, Hungary; 3 Department of Prosthodontics, Semmelweis University, Budapest, Hungary

**Keywords:** Mouthwashes, Chlorhexidine, Chlorine dioxide, Cytotoxicity, HGEPp, Impedimetry

## Abstract

**Objectives:**

The gingiva epithelium accounts for a significant proportion of the surface around the tooth. An inflammatory reaction occurs in the presence of bacterial biofilm, adhesion is reduced, and the depth of the sulcus gingivalis increases. The most common antiseptic agents in oral rinses are chlorhexidine digluconate (CHX) and cetylpyridinium chloride. We examined long-lasting effects of residual concentrations of eight commercially available rinses. Our main goals were (i) to analyze the effect of different chemical compositions on cell proliferation, (ii) to examine apoptosis, and (iii) cell morphology on human epithelial progenitor cell line (HGEPp).

**Materials and methods:**

Cell proliferation was measured in a real-time system (0–48 h) by impedimetry (xCELLigence). Apoptosis was measured with labeled Annexin-V (BD-FACScalibur).

**Results:**

Changes in proliferation were measured at certain concentrations: (i) H_2_O_2_ proved to be cytotoxic at almost all concentrations; (ii) low concentrations of CHX (0.0001%; 0.0003%) were proliferation inducers, while higher concentrations were cytotoxic; (iii) for ClO_2_, advantageous proliferative effect was observed over a broad concentration range (0.06–6 ppm). In mouthwashes, additives in the formulation (e.g., allantoin) appeared to influence cellular responses positively. Apoptosis marker assay results suggested a low-level activation by the tested agents.

**Conclusions:**

Mouthwashes and their reference compounds proved to have concentration-dependent cytotoxic effects on human gingival epithelial cells.

**Clinical relevance:**

A better understanding of the effects of mouthwashes and their reference compounds is particularly important. These concentration-dependent effects (cytotoxic or proliferation inducing) interfere with human cells physiology while being used in the fight against the pathogenic flora.

**Supplementary Information:**

The online version contains supplementary material available at 10.1007/s00784-022-04422-z.

## Introduction

In line with today’s trends in dentistry, the materials used should be esthetically pleasing as well as they should also represent adequate biocompatibility. The prokaryotic flora of the oral cavity and the patient’s own eukaryotic cells are fundamentally different targets from a cell biological and pathological perspective. Due to their chemical nature, the surface membrane and the cytoplasmic components (e.g., G protein-coupled receptor (GPCR) and signaling pathways) are capable of drug-specific perturbations of the cell [1]. Mouthwashes are most commonly encountered by patients at home, for their antibacterial effects so it is especially important to know their effects due to the uncontrolled conditions in which they are applied [2–4]. Biofilm can be considered as a special tissue formation (constantly changing microbiome) that, due to its complexity, can be reduced by the combined application of mechanical and chemical factors [5, 6]. Fortunately, these interventions mainly affect biofilm pathogens, but the effect of some mouthwash components on the oral epithelium cannot be ruled out [7–9]. The antiseptic nature of mouthwashes also affects prokaryotic commensal bacteria which has a role in the maintenance of oral health [10]. Because of their active ingredients, mouthwashes are involved in reducing the pathogenic flora of the oral cavity [11–14].

Chlorhexidine (CHX) is the most commonly used active ingredient in commercially available mouthwashes. Jenkins et al. [15] conclude that the anti-plaque activity of CHX is more due to the formulation (concentrations and dosage) than only to the concentration of CHX used. The most common side effects are the esthetically undesirable staining of the teeth and taste loss. This, however, can be easily resolved with some water rinsing after the use of the mouthwash, or choosing a mouthwash with less CHX or none at all [16–18].

Hydrogen peroxide (H_2_O_2_) is one of the disinfection compounds that has been in use for the longest. In dentistry, it has been used for tooth whitening and for its antiseptic nature, as a result of its oxidizing powers [19, 20]. In higher concentrations, H_2_O_2_ has an immediate toxic effect (with a wide-range damaging effect on lipids, DNA, and proteins), while in lower concentrations, it can induce apoptosis through the activation of the mitochondrial pathway [21]. In recent years, the use of higher concentrations of H_2_O_2_ has been discouraged, as it damages the process of wound healing by, e.g., scratching assay [22].

Chlorine dioxide (ClO_2_) has a size-selective antimicrobial effect not only on bacteria but also on viruses. This chemical not only attacks the cell membrane and cytoplasmic proteins through amino acids (Tyr, Cys, Trp, Met, and Gln), but can also react with cations such as Mn^2+^ and Fe^2+^. Because of the special target mechanism, the microbe does not have the ability to develop resistance against ClO_2_. This ability to only react with the substances listed above makes it possible for ClO_2_ to be also effective in polluted environment [23, 24].

A new way of production (which results in a super-pure ClO_2_) made it possible for ClO_2_ to be used as a highly active disinfectant agent [25]. The essence of its mechanism of action is that the critical exposure time increases with the square of the characteristic size of the target cell [23]. The degradation of this new, high-purity ClO_2_ takes only a few minutes, thus not dangerous for eukaryotic cells. Bacterial cells and viruses being much smaller are therefore in danger of the antiseptic effects of ClO_2_.

Cetylpyridinium chloride (CPC) is a broad-spectrum antimicrobial compound frequently used in dentistry. It is often used as an active ingredient in mouthwashes on its own or in combination with CHX [26]. Other than preventing the formation of new bacterial biofilm, it can prevent pathogen bacteria from releasing pro-inflammatory agents (e.g., IL-1b, IL-8, TNF-α), thus reducing bleeding of the gingiva [27, 28]. The side effects of CPC are more infrequent and are quicker to disappear with the discontinuation of use than those of CHX. In dentistry, CHX on its own—in higher concentrations—is advised to be used only for a short period of time as an acute cure (up to a maximum of two weeks), while CPC with its less effectiveness could be used every day for longer periods [29, 30].

A novel way of the characterization of mouthwash-induced cytotoxicity on human gingival epithelial progenitor cells was applied using real-time measurements with impedimetry (Real-Time Cell Analysis – RTCA). The measured indices are based on the cell physiological responsiveness of model cells, composing tissue elements in the oral cavity. Human gingival epithelial progenitor cells are of great importance since they make up the majority of tissue surrounding the teeth; thus, they are likely to come into contact with materials used in different fields of dentistry. The main goal of our investigation was to contribute data with a novel technique to better understand the long-lasting cell physiological effects of residual amounts of mouthwashes on human gingival epithelial progenitor model cells (HGEPp). Our aims were as follows: (i) How do rinsing agents with different chemical compositions and their active ingredients affect the viability and proliferation of human gingival epithelial progenitor cells (HGEPp)? (ii) Could apoptotic mechanisms be assumed in the case of cytotoxicity induced cell death? (iii) Do the tested substances/mouthwashes cause cell morphological deviations detectable by computer-assisted morphometry in the tested eukaryotic oral cell line? (iv) Are the additional components responsible for some cytotoxic effects?

## Material and methods

The reference compounds CHX and H_2_O_2_ were acquired from the Central Pharmacy Department of Semmelweis University, while CPC was obtained from Sigma Ltd. (St. Louis, USA). High-purity ClO_2_ (Solumium™, Solumium Ltd., Hungary) was prepared by a novel membrane technology [23] at the Department of Physics, Budapest University of Technology and Economics. More detailed description of the technology cannot be given due to patent protection [25]. The composition of commercially available mouthwashes used in our experiments is shown in Table 1. These mouthwash samples were obtained from Sanitaria Kft (Budapest, Hungary). Different dilution protocols were used for reference compounds and the commercially available mouthwashes. For reference compounds, the actual concentrations are presented in “%,” while when discussing the commercially available mouthwashes, the values are in “% v/v,” given that these solutions have numerous ingredients. For dilutions and cell proliferation, CnT-24 medium (CELLnTEC, Bern, Switzerland) containing recombinant materials were used. In concentration course experiments, the dilutions were in correspondence with each substance in clinical use. In reference compounds, the following ranges were used: CHX – 0.0001 – 0.1%; H_2_O_2_ – 0.0003 – 6%; ClO_2_ – 0.06 – 60 ppm; CPC – 0.0005 – 5%. In commercially available compounds, 2E − 07 – 0.2%v/v dilutions were used. These concentrations were more concentrated or much more diluted than those recommended by the manufacturer. Low concentrations model the decreasing post-use concentrations left over from residual substances in the oral cavity. These can remain in the oral cavity for up to several hours after the recommended 1–2 min of use. This long-term effect was assayed using incubation times up to 48 h (72-h incubation was exclusively required in Listerine products because of their alcohol content) [31–33]. Concentration ranges of the tested compounds were always prepared right before experiments.

**Table 1:** Composition of the main ingredients in tested commercially available mouthwashes

Mouthwashes – Commercial name	Main ingredients of the product specified by the manufacturers				
	Chlorhexidine (CHX) (%)	Cetylpyridinium chloride (CPC) (%)	NaF (ppm)	Xylitol (%)	Other
Gum Paroex	0.12	0.05			Vitamin E
Perio Aid 0.12%	0.12	0.05		1	
Perio Aid Maintenance	0.05	0.05		1	
Vitis Gingival		0.05		1	Provitamin B5, zinc lactate
Vitis Orthodontic		0.05	226	1	Vitamin E, allantoin, aloe vera
Dentaid Xeros			226	3.30	Allantoin, betaine
Listerine Fluoride Plus			450	1	Thymol, menthol, methyl salicylate, eucalyptol
Listerine Cool Mint					Thymol, menthol, methyl salicylate, eucalyptol

### Model cell

Assays were performed on primary non-neoplastic, monolayer cultures of pooled human gingiva epithelial progenitor (HGEPp) cells, vial containing > 5 × 10^5^ viable cells/1 ml (CELLnTEC, Bern, Switzerland). HGEPp cells were cultivated in CnT-24 medium (CELLnTEC) containing recombinant additives for cell proliferation. The cell cultivation followed the classical protocol with the addition of 1–1% penicillin/streptomycin (Invitrogen) and L-glutamine (Invitrogen) to the medium. After washing with PBS, 0.25% Trypsin–EDTA solution (Thermo Fisher Scientific) was used to release adherent cells from the culture vessel surface into suspension. To determine the optimal confluency of cultures for successful passages, live fluorescent cell movie analyzer JuLI FL (Nano Entek) was used. A confluence level of 70% of the cultures was considered adequate for passage. Cells from P4-P5 passages were used for the experiments. These passage numbers allowed for the study of relatively young cells. Further information of the cell culture is available on the datasheets of the manufacturer CELLnTEC [34, 35]

### Cytotoxicity–impedimetry

The xCELLigence Real-Time Cell Analysis SP (ACEA Biosciences) instrument was used to monitor the cytotoxic effects of the mouthwashes. In the assay system, gold electrodes are located at the bottom of each well of the 96-well array (E-plate). In the case of arrays connected to an AC system, the adhesive cells form an insulating layer on the gold electrode. Due to the electrical insulating property of the surface membrane of the living cell, an impedance signal (Z) is generated. The magnitude of this signal is increased in proportion to the number of adherent cells, making it suitable for tracking the number of viable and adherent cells. This instrument allows for real-time measurements with a sampling frequency of up to 15 s, as well as cell proliferation. Cell-free wells (loaded with pure fresh medium) were used as cell-free control. These plateau phases are not shown in the cytotoxicity figures. Cells were loaded into the arrays (10^4^ cell/well), and no treatment was given in the first period (60 min) of the experiment. After the initial proliferation phase—when the cells reached their plateau phase, forming a monolayer—, the test compound was loaded, and the impedimetric changes were recorded. (The monolayer formed from 10^4^ cells/well allows registration of both a decreased impedimetric signal (in cytotoxicity) and increased impedimetric signal (in proliferation). From the change in impedance obtained during the measurements, the Cell Index (CI) can be calculated using the following formula:

1 $$\mathrm{Cell}\;{\mathrm{Index}}_{\mathrm i}=\left({\mathrm{Zt}}_{\mathrm n}-{\mathrm{Zt}}_0\right)/{\mathrm F}_{\mathrm i}$$

In the formula, Zt_n_ is the impedance measured at the given time, while Zt_0_ is the impedance measured at the starting time; *F*_i_ is the frequency constant, which is 15 in this case. Data normalized over the time (Delta Cell Index) were used to evaluate effects of treatments.

Cell viability (toxic effect of substances) was measured for 24, 48, and, in some cases, 72 h. The change in impedance was measured by the device every 60 s. During the experiments, the statistical calculations were performed by averaging the results of at least 4 × 3 parallel (n = 12) measurements [23].

**Fig. 1:**
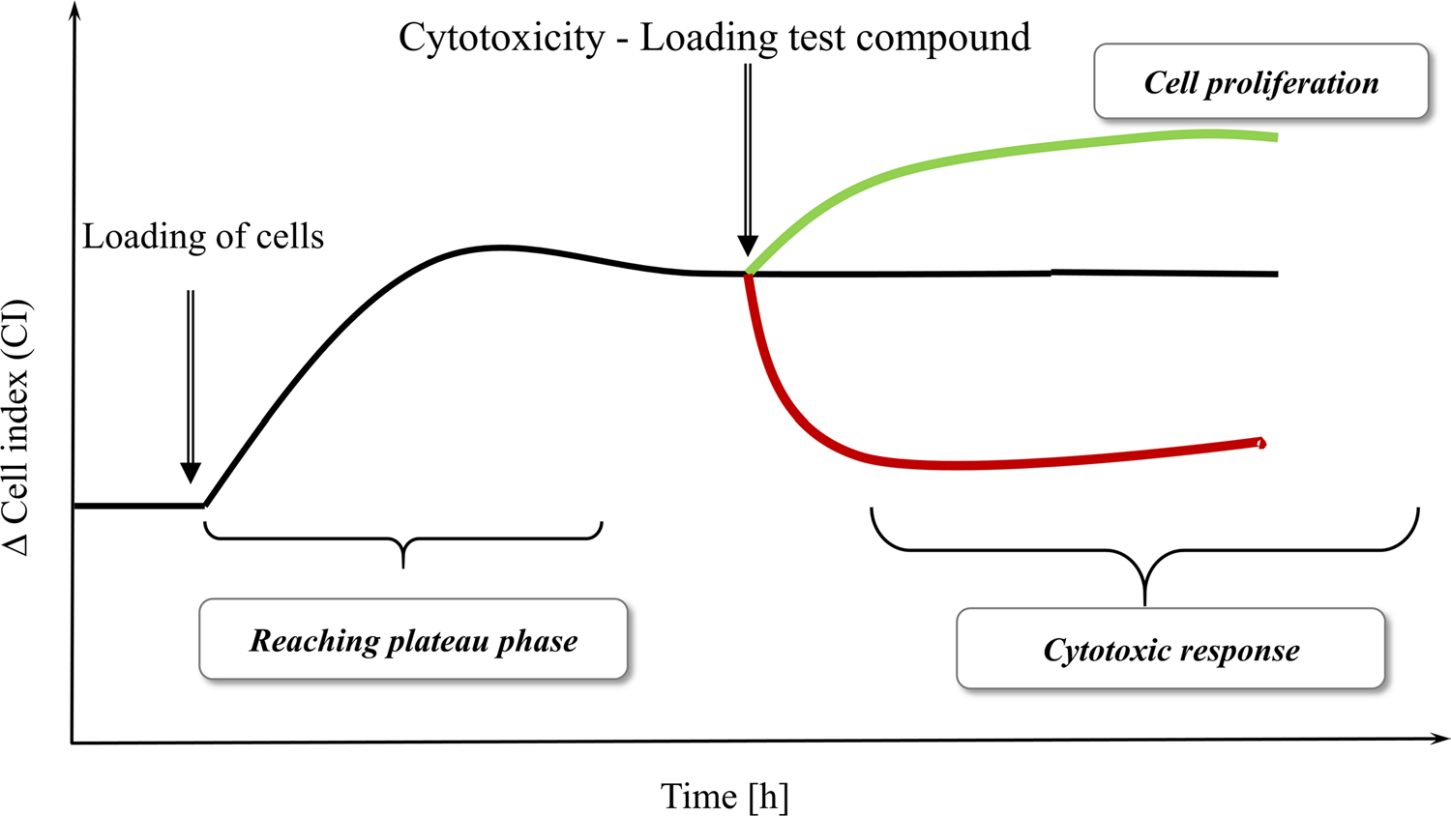
Understanding the changes of impedance to measure cytotoxicity/cell proliferation

As shown in Fig. 1, a change in the impedance signal is relevant with regards to the nature of the test substance (decreases in the case of cytotoxicity, increases in the case of proliferation). In the initial phase of the impedance or CI curves, transient peaks are observed due to the loading of the cells in the E-plates (e.g., Fig. 3a, b). These are transient (max. 10–20 min) artifacts, independent of the effect of the test substance, resulting from the used technique. The causal effect does not influence the cell biological interpretability of the measurements when evaluating the curves. The half maximum inhibitory concentrations (IC50) were calculated to further characterize cytotoxicity changes within 24 and 48 h. The ratio of the two values — IC50(48 h)/IC50(24 h) — was also calculated to measure the dynamics of cytotoxicity elicited by the compounds.

### Apoptosis assay

For the study of apoptosis, an early apoptosis marker, the phosphatidylserine-externalization, was detected by using Annexin V. The membrane asymmetry characteristics of living cells are overturned during apoptosis, and only the inner membrane-specific phosphatidylserine is deposited in the outer layer of the surface membrane. Due to the displacement, the labeled annexin V — applied extracellularly — is able to bind to phosphatidylserine. To detect phosphatidylserine expressed on the surface of apoptotic cells, an Annexin V-PE Plus (MBL International) apoptosis detection kit was used. During the assay, the cells to be tested (5 × 10^4^ cells/sample) were treated with solutions of mouthwashes that proved to be cytotoxic in our pilot experiments for 48 h. (By the end of 48 h, the effects elicited by the compounds did not change characteristically). The incubation with mouthwashes or active ingredients was followed by washing in PBS and centrifugation (1000 rpm/5 min). After removing the supernatant, the sample containing cells was resuspended in 300 µl assay buffer, after which 3 µ l of Annexin V-PE and 0.6 µ l of SYTOX Green dye (Invitrogen) were added to the cells. Incubation was performed at room temperature, protected from light, for an incubation time of 10 min. Annexin V-positive cells were evaluated with flow cytometry BD FACSCalibur (BD Biosciences) (Annexin V-PE: Ex = 488 nm, Em = 578 nm; SYTOX: Ex = 488 nm, Em = 525 nm) and under fluorescent cell movie analyzer JuLI FL (Nano Entek). Data acquired were then analyzed by CellQuest Pro software.

### Computer-based morphometric analysis

Treatment with mouthwashes also caused visible changes in the morphological parameters of HGEPp cells. The morphological changes elicited by the 48-h treatments were recorded on native microscopic samples, and 50 × magnification images of a Zeiss Axiovert A1-inverted microscope (Carl Zeiss) were used for these studies. Computer-assisted morphometric analysis was performed using video recordings (5 recordings/group). HGEPp cells were incubated for 48 h with test compounds. 200 µl of the samples were placed onto predegreased surfaces. Each (video recorded) frame contained approximately 10–12 cells/microscopic fields for 120 s at a maximum frame rate of 3 frames/s. For this purpose, we used the Biomorph 1.1 program developed by Chemotaxis Research Group, GCI-SU [36]. This morphometry analysis performed the cluster analysis of the data in addition to the basic morphometric parameters (Area, Perimeter) of the examined cells.

### Statistical evaluation

In our studies, a minimum of four parallel measurements with three replicas (*n* = 12) were performed in each case. For impedimetric studies, the slope analysis of the obtained curves was calculated by the xCELLigence SP equipment’s statistical program (RTCA 2.0, Real Time Cell Analyzer; ACEA Biosciences). For the statistical evaluations of the obtained results, one-sample ANOVA and the statistical routines of Origin Pro 8.0 were used. The following symbols are used to denote the levels of significance: *z* ≤ 0.005, *y* ≤ 0.01, and *x* ≤ 0.05. To determine the IC50 values, we used a fitting sigmoidal dose response curve with the nonlinear regression function of the Origin Pro8.0 program (OriginLab) based on the following equation:

2 $$y={A}_{2}+\left({A}_{1}-{A}_{2}\right)/\left(l+exp\left(\left(x-{x}_{0}/dx\right)\right)\right)$$

For the morphometric evaluation, the built-in statistical routines of Biomorph 1.1 were used.

## Results

### Cytotoxicity–impedimetry

The cell physiological responses elicited by the dental substances were monitored by impedimetry. The responses are explained using the impedimetric curve profile (Fig. 1). In the case of cytotoxicity or proliferative effects, cells that were loaded previously and are in the plateau phase of growth are treated with the test compounds. The registered signal indicates the cytotoxic or proliferative effects. In the pilot experiments, some high concentrations (0.2 and 0.4%v/v) of mouthwashes and their active ingredients proved to be unusable in the cell physiology assays, due to their precipitation in the solvent. Therefore, these precipitating concentrations were omitted from evaluation (Table S1). In general, the analysis of the obtained impedimetric curves shows that fundamentally different curve characteristics were observed for the four reference compounds (see below). In the case of commercially available mouthwashes — except for the significant cytotoxic effects elicited in high concentrations—the effects mostly did not deviate from the control during the first 20 h of the treatment. After 20 h of incubation, and depending on the nature of each substance, a cell proliferation-increasing effect could be recorded at lower concentrations.

### Reference compounds

#### Hydrogen peroxide

**Fig. 2:**
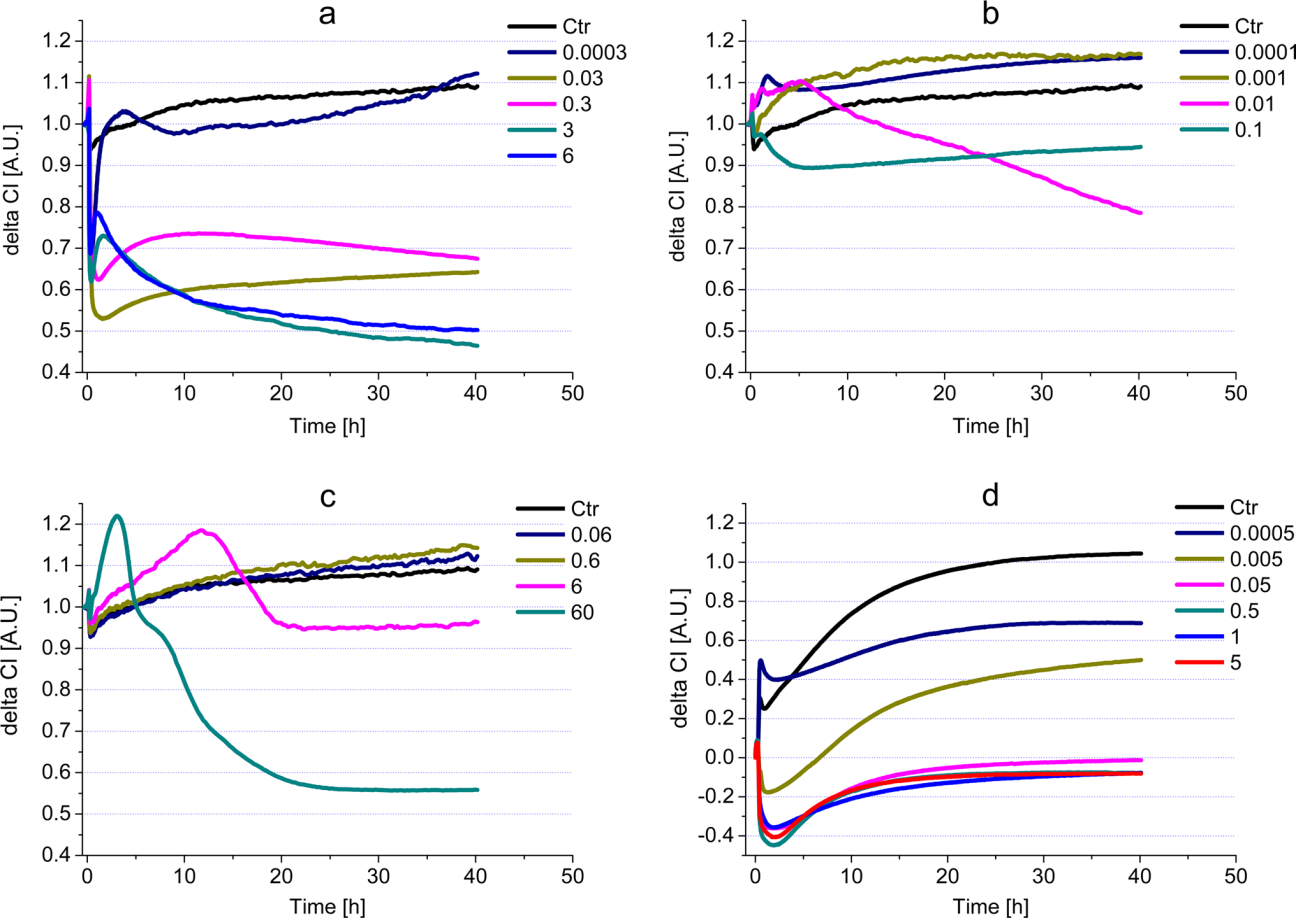
Impedimetric analysis of cell viability modulated by the most common active ingredients (**a** H_2_O_2_, **b** CHX, **c** ClO_2_, **d** CPC) in HGEPp cells

At the start of the experiment, we see signal peaks, but these peaks are considered as artifacts caused by inserting and/or removing the E-plates. The concentrations applied had an immediate effect resulting in the decreased viability of the cells (Fig. 2a). This is visible from the decreased impedance signal and, in the case of concentrations 6% and 3%, the signals run low throughout the experiment. In the case of 0.3% and 0.03%, the immediate toxic effect was followed by a weak increase, but both remained intensely cytotoxic. The lowest measured concentration was 0.003%; its value remained close to the control line, implying that it was neither cytotoxic, nor did it influence the proliferation of the HGEPp cells.

As seen in Table 2, the IC50 values of H_2_O_2_ show that cells lose viability developed in the first 24 h (0.027) without significant change at 48 h (0.028).

**Table 2:** Comparison of IC50 values of reference compounds and commercially available mouthwashes

Active ingredients	Maximal non-toxic	IC50 (24 h)	IC50 (48 h)	Unit	IC50 (48 h)/IC50 (24 h) ratio
CHX	0.001	0.01	0.009	%	0.900
H_2_O_2_	0.0003	0.027	0.028	%	1.037
ClO_2_	0.6	20.40	20.51	ppm	1.005
CPC	*	0.003	0.003	%	1.000
Commercially available mouthwashes					
Gum Paroex	0.0002	0.002	0.0015	%v/v	0.75
Perio Aid 0.12%	0.0002	0.04	0.005	%v/v	0.125
Perio Aid Maintenance	0.0002	0.004	0.001	%v/v	0.25
Vitis Gingival	0.0002	0.01	0.001	%v/v	0.1
Vitis Orthodontic	0.0002	0.01	0.005	%v/v	0.5
Dentaid Xeros	0.02	0.069	0.063	%v/v	0.913
Listerine Fluoride Plus	0.00002	0.005	0.002	%v/v	0.4
Listerine Cool Mint	0.002	0.01	0.01	%v/v	1.0

^*^In the case of CPC, all the measured concentrations had a significantly lower delta CI than the control, implying that the concentrations measured in our experiment were all toxic to the model cells

*ND* not detectable cytotoxicity

#### Chlorhexidine

The most concentrated solution (0.1%) of CHX elicited an immediate decrease in the impedimetric signal, which remained persistent, implying an intense cytotoxic effect (Fig. 2b). The 0.01% solution had a transiently increased impedimetric signal, surpassing the control line and after the 10th hour it turned into a steady decrease. Around 25 h, the 0.01% and 0.1% lines cross which implies that in the long run (> 25 h) 0.01% concentration CHX can express a stronger cytotoxic effect than 0.1% CHX. The two lowest concentrations (0.001% and 0.0001%) of CHX surpassed the control line significantly for the entire experiment. The concentrations used in the experiment were more diluted than those used in commercially available mouthwashes, or the concentrations used for therapeutic purposes in dental practices (0.2% and 0.12%). The 24-h IC50 value (0.01) shows that CHX had a strong cytotoxic effect on the epithelial model cells, and this effect did not change significantly for the rest of the experiment (IC50 (48 h) was 0.009). (Table 2).

#### ClO_2_

In our experiment, the most concentrated solution of ClO_2_ was 60 ppm, which is more concentrated than the therapeutical recommendation by the manufacturer [37] (Fig. 2c). This 60 ppm ClO_2_ caused a rapid increase of impedimetric signals in the first hours of the experiment (2–3 h). However, this increase turned into a deep dive and remained toxic for the rest of the experiment. The 6 ppm ClO_2_ had an almost identical effect on the above-mentioned concentration, with the difference that the 6 ppm solution had an elongated increase and decrease (reaching its peak at ~ 13 h), which plateaued under the control line. The 0.6 ppm and 0.06 ppm solutions had similar impedimetric signals to the control line. Although a slight increase (from 21st h) was detected, it did not significantly differ from the control line. The IC50s for 24-h and 48-h incubations were similar (20.40 ppm and 20.51 ppm, respectively) suggesting that the 24-h incubation was enough to achieve the maximum decrease in cell viability (Table 2).

#### CPC

The four highest tested concentrations (0.05%, 0.5%, 1%, and 5%) had very similar profiles of impedimetric curves (Fig. 2d). These concentrations of CPC induced a prompt drop in the impedimetric values, which turned into steadily increasing impedimetric values, resulting in plateaus. However, these concentrations were intensely toxic to HGEPp cells throughout the entire incubation time. The impedimetric profile of 0.005% had a similar character to the previously mentioned highest concentrations, with the difference that it plateaued higher. The 0.0005% CPC caused a similar viability decreasing effect; nevertheless, this concentration of CPC was the closest to the control among the tested CPC concentrations, but it still remained in the toxic range.

The calculated IC50 values for 24 h and 48 h were 0.003%, which shows that CPC reaches its maximum toxicity within 24 h (Table 2).

### Commercially available mouthwashes

In contrast to reference compounds, the tested commercially available mouthwashes showed four characteristic patterns of concentration dependence (i–iv) of cytotoxicity, displayed in Table 3. (i) Some concentrations caused an immediate, intense and long-lasting cell viability decreasing effect. (ii) In some cases, the compounds were still cytotoxic, but had a transient increase that turned into a steady decrease for the rest of the incubation. (iii) Some of the tested concentrations of the mouthwashes elicited an increased (moderate or weak) proliferation-inducing behavior. (iv) Several concentrations of the mouthwashes had no significant effect on cell viability or proliferation of the model cells.

**Table 3:** Concentration-dependent, significant cell physiological effects (i–iv) elicited by mouthwashes in HGEPp cells

	Cytotoxic: intense, steady (i) [%v/v]	Cytotoxic: with transient increase (ii) [%v/v]	Proliferative (iii) [%v/v]	Neutral (iv) [%v/v]
Gum Paroex	0.02^z^	0.002^y^		0.0002–2E − 07
Perio Aid 0.12%	0.02^z^	0.002^z^	0.0002^z^, 2E − 05^y^, 2E − 06^z^	6.67E − 05, 2E − 07
Perio Aid Maintenance	0.02^z^, 0.002^y^		*0*–*20 h* 6.67E − 05^z^, 2E − 05^z^, 2E − 07^z^	*0*–*20 h* 0.0002–2E − 07 *20*–*55 h* 0.0002, 2E − 06
Vitis Gingival	0.02^z^	0.002^y^	*20*–*55 h* 0.0002^y^	*0*–*20 h* 2E − 07–0.0002
Vitis Orthodontic	0.02^z^	0.002^y^	*20*–*55 h* 0.0002^z^, 2E − 06^y^	2E − 07, 2E − 05, 6.67E − 05
Dentaid Xeros	0.2^z^		6.67E − 05^y^, 2E − 06^y^	0.02–0.0002, 2E − 05, 2E − 07
Listerine Cool Mint	0.2^z^, 0.02^y^	2E − 07^x^ *w/o decrease		0.002–2E − 06
Listerine Fluoride Plus	0.2^z^, 0.02^z^	0.002^y^, 0.0002^x^ *w/o decrease	2E − 07^z^	6.67E − 05–2E − 06

(x – p < 0.05; y– p < 0.01; z – p < 0.001)

#### Mouthwashes containing CHX and CPC

**Fig. 3:**
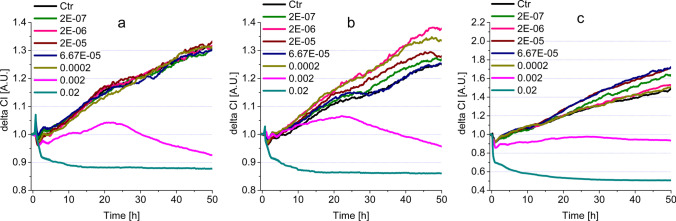
Impedimetric analysis of cell viability modulated by commercially available mouthwashes, containing both CHX and CPC, on HGEPp cells (**a** Gum paroex; **b** Perio Aid 0.12; **c** Perio Aid Maintenance)

This group of mouthwashes showed intense steady or transient cytotoxic character at high concentrations (0.02, 0.002%v/v), (Fig. 3 and Table 3). The impedimetric assays show that strong cytotoxicity was detectable for all three rinsing agents (Gum Paroex, Perio Aid 0.12 and Perio Aid Maintenance) in this group. However, in some cases (Gum Paroex – Fig. 3b and Perio Aid 0.12 – Fig. 3c, Table 3 (ii)), a transient increase below the control level was also observed. This measurement peaked around 20 h and then a consistent cytotoxic effect was registered around 30 h. An opposite, proliferative effect was also observed at some concentrations. This was observed for Perio Aid 0.12 and Perio Aid Maintenance samples, with the difference that Perio Aid 0.12 showed a stronger proliferative character (Table 3 (iii), Fig. 3b), while the effect of Perio Aid Maintenance turned weaker after 20 h (Table 3 (iii), Fig. 3c). For several concentrations, indicated in Table 3 (iv), no cytoxic or proliferative character was observed.

#### Mouthwashes containing CPC

**Fig. 4:**
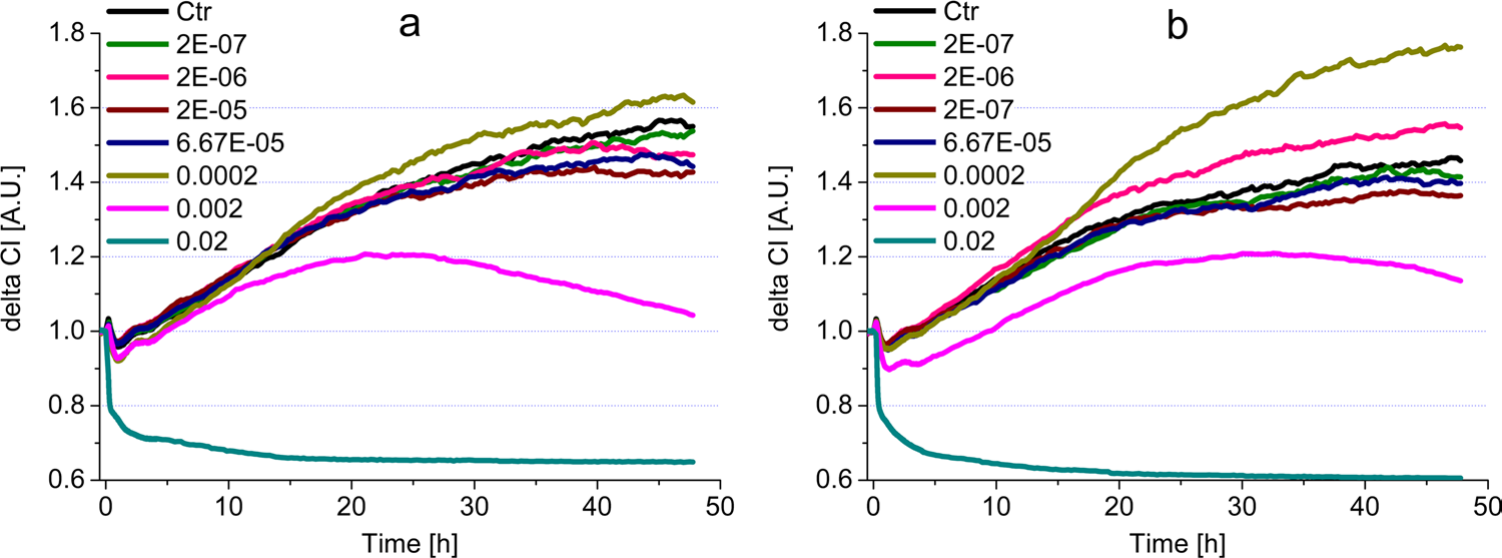
Impedimetric analysis of cytotoxicity modulated by commercially available mouthwashes, containing CPC on HGEPp cells (**a** Vitis Gingival; **b** Vitis Orthodontic)

In the case of mouthwashes containing only CPC as a reference compound (Vitis Gingival and Vitis Orthodontic), a cytotoxic effect was observed similarly at the two highest concentrations (Fig. 4a, b, Table 3 (i)). A transient increase in cell numbers was also observed, with a peak at 25 h for Vitis Gingival and at 30 h for Vitis Orthodontic. Subsequently, in both cases, the given concentration had a gradually increasing toxic effect until the end of the experiment (Fig. 4a, b, Table 3 (ii)). A proliferative effect was observed only at concentrations of 0.0002 and 2E − 06%v/v. Of these, the effect of 0.0002%v/v Vitis Orthodontic was particularly strong (Fig. 4b). However, many of the concentrations tested did not show any cytotoxicity or increase in cell numbers in HGEPp cells (Table 3 (iv)).

#### Mouthwashes containing no reference compounds

**Fig. 5:**
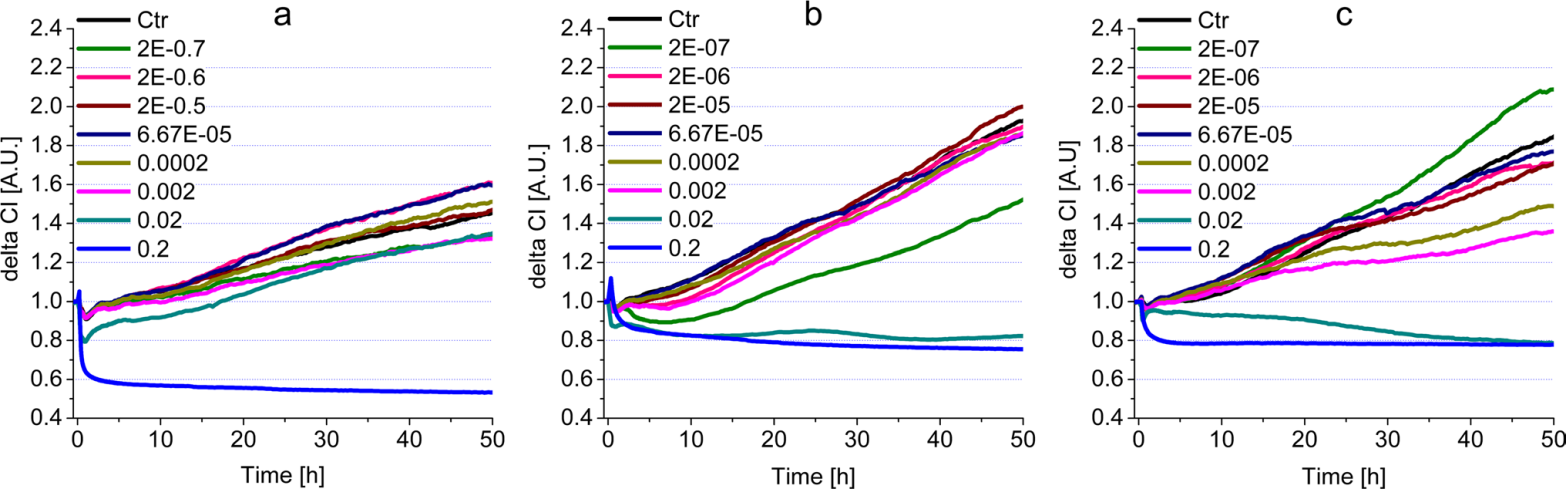
Impedimetric analysis of cytotoxicity modulated by commercially available mouthwashes, containing no reference compounds, on HGEPp cells (**a** Dentaid Xeros; **b** Listerine Cool Mint; **c** Listerine Fluoride Plus)

In the case of mouthwashes containing no reference compounds (Dentaid Xeros, Listerine Cool Mint and Listerine Fluoride Plus), similarly to the other substances discussed earlier, the highest concentrations proved to be cytotoxic (Fig. 5, Table 3 (i)). In the case of Dental Xeros, only 0.02%v/v had cytotoxic effect (Fig. 5a, Table 3 (i)). On the other hand, Listerine Cool Mint and Listerine Fluoride Plus samples showed intense, steady cytotoxicity in two concentrations (0.02 and 0.002%v/v) (Fig. 5b, c, Table 3 (i)). An interesting curve profile was observed for Listerines: 0.002 and 0.0002%v/v Listerine Fluoride Plus (Fig. 5c) and 2E − 07%v/v Listerine Cool Mint (Fig. 5b) resulted in lower run curves than several other concentrations and the control. However, these characters cannot be considered as transient cytotoxic effects, as their course shows a continuous upward trend, parallel to the control. Studies have shown that these samples, which do not contain reference compounds, were also able to increase proliferation at relatively low concentrations (Table 3 (iii)). In addition to the effects described above, we also found a number of concentrations with no positive or negative effect on cell numbers (Table 3 (iv)).

Similarly to the description of the cytotoxic effects of reference compounds, the IC50-based assessment for commercial mouthwashes is accepted in the literature (Table 2). In this case, the comparison of the IC50 values at 24 and 48 h provided valuable data about the dynamics of cytotoxicity. In the case of CHX- and CPC-containing compounds, increased significant cytotoxicity was observed in IC50(48 h)/IC50(24 h) ratios: Perio Aid 0.12%—0.125; Perio Aid Maintenance—0.25. In the CPC-containing solution, Vitis Gingival proved to have a strong, progressive cytotoxicity inducing effect, while Vitis Orthodontic had moderate effect (IC50(48 h)/IC50(24 h) ratios: 0.1 and 0.5 respectively). Mouthwashes containing no reference compounds had no long-term cell viability influencing effects, only Listerine Fluoride Plus elicited a weak effect IC50(48 h)/IC50(24 h) ratio: 0.4.

### Apoptotic effects

**Fig. 6:**
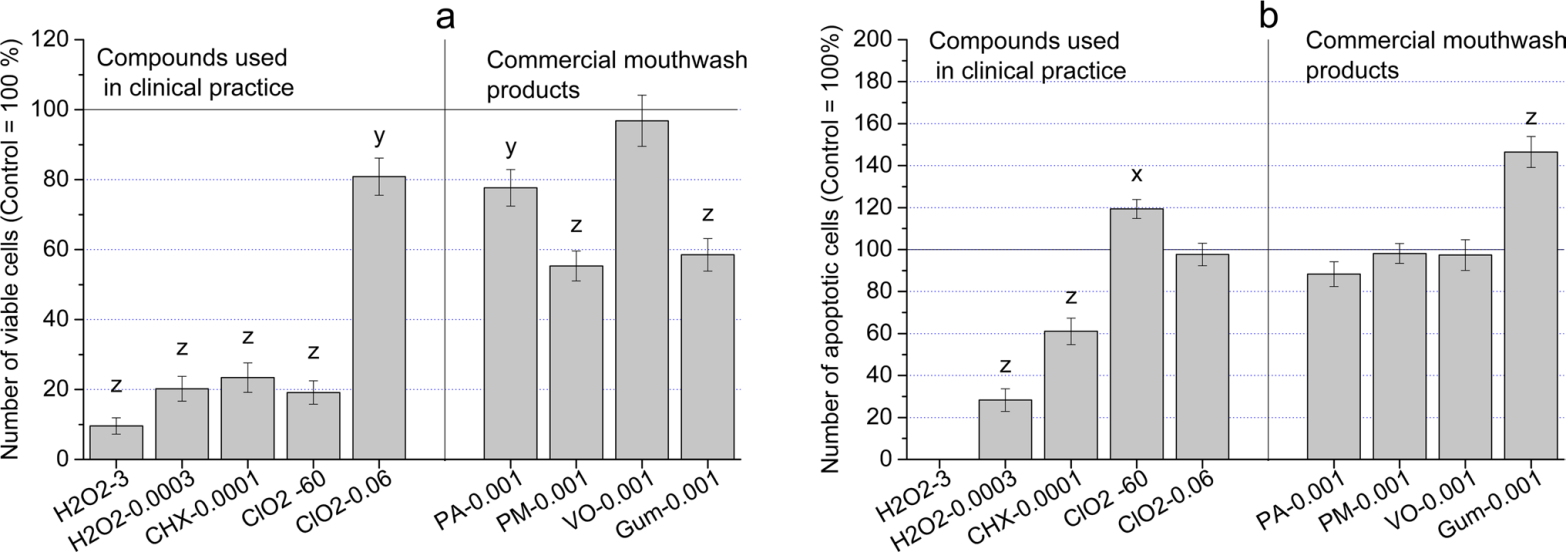
**a** Viability of HGEPp cells treated by H_2_O_2_, CHX, ClO_2_, and commercially available mouthwashes (PA – Perio Aid 0.12, PM – Perio Aid Maintenance, VO – Vitis Orthodontic, Gum – Gum Paroex); **b** Annexin V positivity of HGEPp cells as a marker of apoptosis inducer effect of compounds used in the clinical care of dentistry (H_2_O_2_, CHX, ClO_2_) and commercially available mouthwashes (PA – Perio Aid 0.12, PM – Perio Aid Maintenance, VO – Vitis Orthodontic, Gum – Gum Paroex)

The decreases in living cell numbers (cytotoxicity—measured by the decrease of impedimetric signals) and changes in cell morphology (the appearance of rounded cells in substantial numbers) are the consequences of cell deaths caused by the concentration-dependent effects of mouthwashes. Figure 6a shows the 24-h treatments causing a significant decrease in the proportion of living cells treated with H_2_O_2_, CHX, and high concentration of ClO_2_, as well as in treatments with Perio Aid Maintenance (PM) and Gum Paroex (Gum). In the case of a mouthwash containing no CHX (Vitis Orthodontic – VO), the lower concentration of ClO_2_ (0.06 ppm) and Perio Aid 0.12% (PA) had similar values to the control. The molecular-level understanding of the effects, described above, raises more possibilities, i.e., induction of apoptosis, increased membrane permeability, and inhibition of intracellular target mechanisms. The most likely reason for cell number decreases, described above, is the triggered early apoptosis, which can be detected by the use of an Annexin V assay. Our data show that this type of programmed cell death might be responsible for cell deaths caused by 60 ppm ClO_2_ (119.46% ± 5.2) and 0.001%v/v Gum Paroex – Gum (146.49% ± 7.8) (Fig. 6b). It is worth mentioning that 3% H_2_O_2_ elicits no apoptosis; viability decrease is most likely a direct result of cytotoxicity. (However, there is also the possibility that due to the oxidizing and reducing nature of H_2_O_2_, the fluorescence of PE used to label Annexin cannot be elicited at high concentrations of H_2_O_2_.)

### Morphology and morphometry analysis

**Fig. 7:**
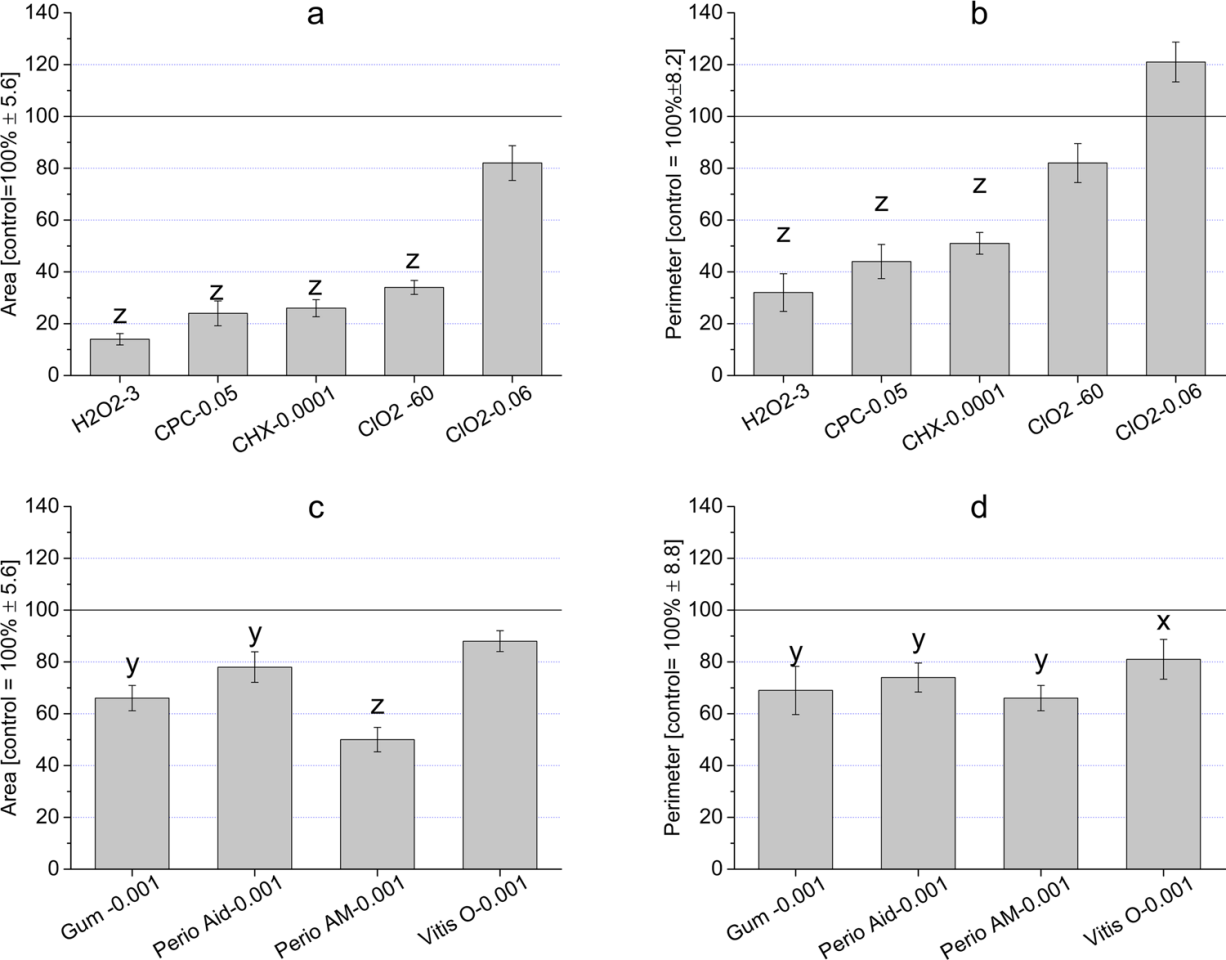
Morphometric changes measured by “Area” (**a**, **c**) and “Perimeter” (**b**, **d**) values in the case of treatments with CHX, H_2_O_2_, ClO_2_, Gum Paroex (Gum), Perio Aid 0.12 (Perio Aid), Perio Aid Maintenance (Perio AM), and Vitis Orthodontic (Vitis O) on HGEPp cells. (Zeiss Axiovert A1 invert microscope 50 × and Biomorph 1.1 program)

The changes in cells caused by the mouthwashes do not only influence their viability, but also affect the morphological characters of surviving and living cells. Changes in morphology were visible at 0.1% and 0.0001% CHX and 3% and 0.0003% H_2_O_2_. The effects of high and low concentrations of CHX, H_2_O_2_, the 60 ppm (0.006%), and 0.06 ppm (0.000006%) ClO_2_ induced the smallest changes to the control (Fig. S1). The 0.05%v/v CPC elicited toxic effects resulting in more rounded cells. These changes were also detectable with a computer-based morphometric evaluation of the indices “Area” and “Perimeter” (Fig. 7). The only concentration which did not cause a characteristic change in cell morphology was the 0.06 ppm ClO_2_. This concentration was neutral to the cells, as it caused no significant change in morphology. If a cell becomes rounded, or its size decreases, it is considered to be the result of some internal regulatory change of mechanism(s).

Even though the cells became more elongated because of treatments with the mouthwashes, it was more of a shrinking effect than a characteristic change in their shape. This change was also visible from the lower numbers of the “Area” value (Fig. 7a). The ruffling characteristics of the cells surface can be indicated by the “Perimeter” value. Ruffled cell surface was detected in the case of treatment with ClO_2_; it resulted in higher “Perimeter” value. Some compounds (CHX and H_2_O_2_) caused cells to become more rounded (Fig. S1), and the computer-based morphometric evaluations are in line with these results. The decreased “Area” and “Perimeter” indices clearly indicate that the cells became roundish and detached from the surface. These are major characteristics of cytotoxic effects and dead cells (Fig. 7b). Unfortunately, separate subpopulations could not be identified by the cluster analysis of Biomorph 1.1.. With regards to the microscopic investigations of cells treated with mouthwashes containing CHX (Gum Paroex, Perio Aid 0,12%, Perio Aid Maintenance), data showed similar morphological changes seen in treatments with CHX on its own. In the case of the mouthwash-free CHX (Vitis Orthodontic), cell morphology did not differ from control cells (Fig. S2). The mouthwashes caused a significant change in the morphometric parameters of model cells (“Area” and “Perimeter”) (Fig. 7c, d). As mentioned above, the roundness of the cells was measured by their “Area” value. Perio Aid Maintenence, Gum Paroex, and Perio Aid 0.12% (all containing CHX) significantly reduced their “Area” values. Even though the “Area” value of Vitis Orthodontic (this mouthwash does not have CHX as one of its ingredients) did not change significantly, “Perimeter” values suffered a significant decrease in every mouthwash tested.

## Discussion

The evaluation of the effect of substances used in dentistry is essential both theoretically and in clinical approaches, too. The cell physiological responses of normal and pathogenic oral flora are crucial; however, in our present study, the main objective was to investigate the response(s) of healthy human gingival epithelial progenitor cells (HGEPp). The different compositions of mouthwashes tested had diverse effects on the model cells. The reduction of cell number (cytotoxicity) and changes in characteristic markers (apoptosis, cell morphology) of decreased cell viability were also considered pathological. In the case of reference compounds (H_2_O_2_, CHX, ClO_2_, CPC), which are known to have cytotoxic effect on pathogen bacteria (which is in correspondence with their clinical usage), intense cytotoxic effects were also observed in many cases on human gingival epithelial progenitor cells throughout experiments (Table S2-A).

Detecting cytotoxic effects and their intensity provided important information for us on mapping adverse side effects (while keeping in mind that in some cases an increase in proliferation is not advantageous) (Table S2A-2B and S3).

The investigated reference compounds (H_2_O_2_, CHX, ClO_2_, CPC) proved to elicit intense and long-lasting cytotoxic effects where H_2_O_2_ and CPC had a wide range of effectiveness (Table S2-A). The concentration dependence of intense responses induced by CHX and ClO_2_ was narrower. The characteristic IC50 24-h and IC50 48-h values of these compounds did not change significantly with the passing of time from the 24th hour to the 48th hour (Table 2). Evaluation of our data pointed out that HGEPp cells express high sensibility to the reference compounds, while the fight against the pathogen flora requires higher concentrations to be effective.

In products where CHX and/or CPC are present as significant components (Gum Paroex, Perio Aid 0.12, Perio Aid Maintenance, Vitis Gingival, Vitis Orthodontic), the cytotoxic character was expressed in lower concentrations (0.02–0.002%v/v) and in full courses of incubation times (0-–5 h). This clinically non-advantageous character was recorded even in cases when some additional ingredients (e.g., NaF, allantoin, xylitol, vitamins) were present. In contrast, in the case of products where the CHX and /or CPC ingredients were not present (Dentaid Xeros, Listerine Cool Mint, Listerine Fluoride Plus) but a list of selected additives enriched the mouthwash, the intense cytotoxic effects were elicited only in higher concentration ranges (0.2–0.02%v/v). (Lower concentrations could elicit effects only in shifted time scales.) The responsible elements in these cases could be NaF in Dentaid Xerose and Listerine Fluoride Plus and the alcoholic component of the two Listerines. The registered proliferative responses were rather sporadic, only Perio Aid 0.12, Vitis Orthodontic, and Listerine Fluoride Plus had intense proliferative nature in HGEPp cell cultures. In these cases, the gradual growth of intensity was registered which reached the real proliferative character only in the late phase of time courses (Table S3). For the significance of additional components, in the cases discussed above, the presumed biological effects of the additional ingredients (cytotoxicity and/or proliferation) have been raised several times. Many ingredients listed in the commercially available mouthwashes are described in the literature as having anti-proliferative (antitumor) effects. These additional components include thymol, NaF, allantoin, Vitamin E, and Aloe Vera. As shown in the Table 4, a significant proportion of these substances are able to exert an inhibitory effect on cell division through various mechanisms. Based on these facts, we assume that in the background of our results, these ingredients may have an important role in the effects on the model cells.

**Table 4:** Cell physiological effects elicited by additional ingredients of commercially available mouthwashes

	Cytotoxic	Ref	Proliferative	Ref	Other	Ref
Thymol	Anticancer	38			Antiapoptotic	39
	Antioxidant	40				
NaF	G2/M cell cycle arrest	41	Proliferation inducer	41	Migration inducer	41
Allantoin	Proliferation inhibitor	42	Proliferation inducer	42	Wound healing	43
Vitamin E	SubG0 cell cycle arrest	44			Proapoptotic	45
Aloe Vera					Wound healing	46
Menthol	Proliferation inhibitor	47			Motility inducer	47
Methyl salicylate	Proliferation inhibitor	48				
Eukalyptol	Oxydative DNA damage inducer	49				

**Fig. 8:**
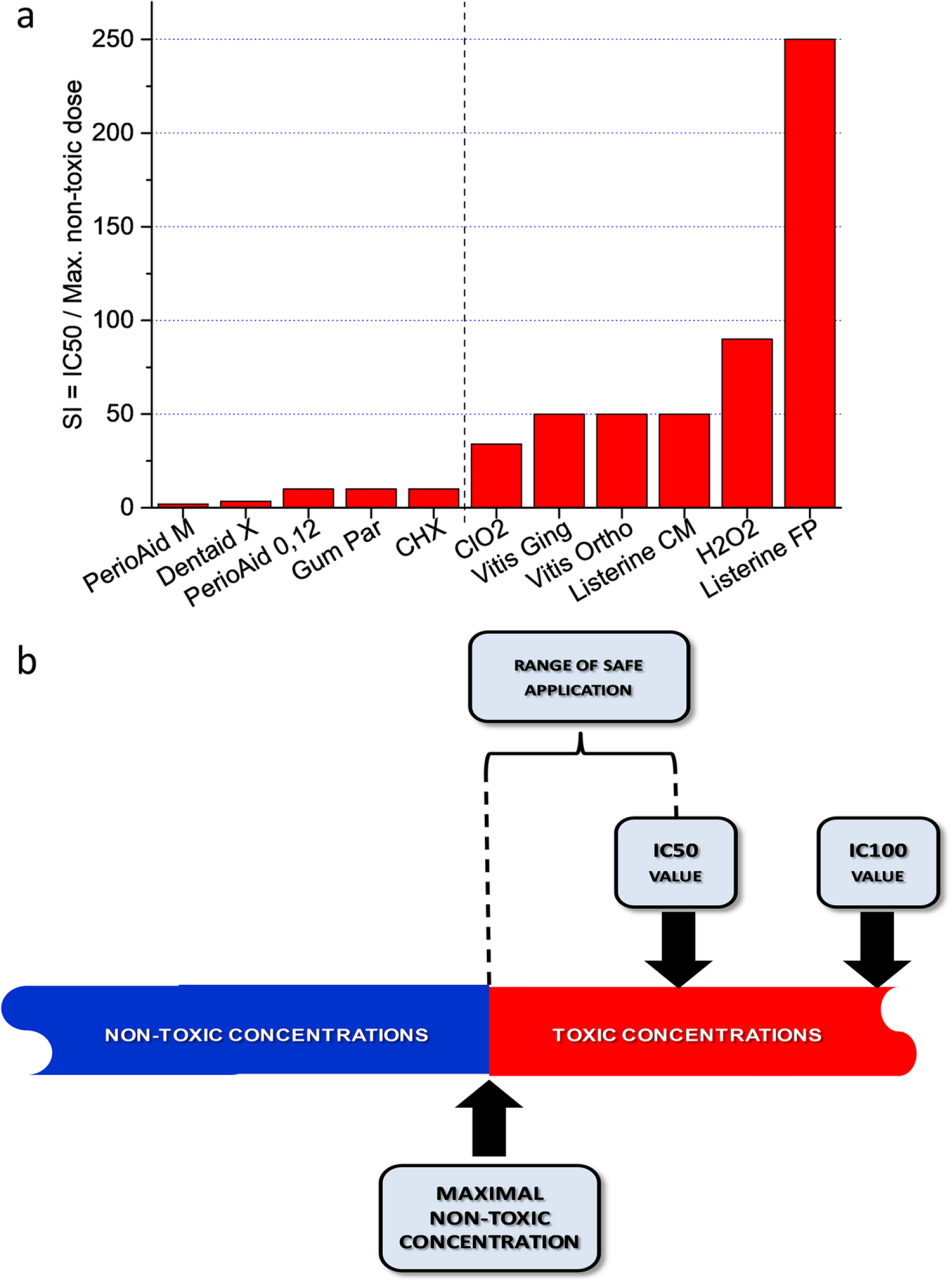
**a** Characterization of safe applicability taking into account IC50/maximum non-toxic concentration ratios (adjusted Safety Index – SI) based on viability index values for HGEPp epithelial progenitor cells; **b** Interpretation of safe applicability taking into account IC50 and maximum non-toxic concentrations

The significance of the IC50 values, in light of the safe use of mouthwashes, our in vitro results showed that the concentration-dependent effect of each substance provided an opportunity to give an accurate characterization of its cytotoxic/cell viability modulator nature by determining the maximum non-toxic concentrations and IC50 values, knowing these two values (or their quotients) can be very important for clinical applications, especially for substances such as mouthwashes, which are used not only in dental practice (see CHX or CPC) but became a part of everyday life due to the need for the maintenance of oral hygiene. As shown in the figures, the range between the maximum non-toxic concentration and IC50 values are not indifferent to the preservation of certain tissue elements in the oral cavity (Fig. 8a, b).

The results of materials studied in the present work show that these materials affect the viability of the gingival epithelium in a concentration-dependent manner. Using the two variables (IC50 value and Maximum non-toxic concentration) described above, our evaluation shows that in this correlation, the high numerical values of the ratio characterize safe compounds in practice, while low values represent a need for a more cautious usage (Fig. 8b). The most ideal substances are the ones where IC50 and maximal non-toxic values are the closest to each other (see adjusted Safety Index = SI) [50]. In our case, the substance meeting the above-mentioned conditions is ClO_2_ (SI = 34). (In the case of Perio Aid Maintenance, the IC50 value is much smaller than the Maximum non-toxic value resulting in a low rate – adjusted SI = 2, while at the other end of the spectrum, with Listerine Fluoride Plus, IC50 value is much greater than the Maximal non-toxic value – adjusted SI = 250.)

3 $$\mathrm{SI}\approx\frac{2\mathrm x\left({\mathrm{IC}}_{50\mathrm n}-{\mathrm{IC}}_{50\mathrm c}\right)}{{\mathrm{TR}}_{\mathrm n}+{\mathrm{TR}}_{\mathrm c}}$$

where SI denotes safety index; IC_50n_ denotes the half maximum inhibitory concentration of non-toxic compound, IC_50t_ denotes the half maximum inhibitory concentration of the toxic compound; TR denotes the therapeutic range for the non-toxic compound (*n*), and for the toxic compound (*t*).

In addition to the direct cytotoxic effects referred to in the literature (e.g., cell cycle arrest, antioxidant effect, oxidative DNA damage induction), the cell number-reducing effects of our tested substances suggest that early apoptosis induction may also occur. Our studies showed that this mechanism was detectable only in 60 ppm ClO_2_ and 0.001%v/v Gum Paroex among the tested substances and concentrations (Fig. 6b). Analysis of the results shows that in terms of viability (Fig. 6a), 60 ppm ClO_2_ has a significantly better value than the other reference substances. (A comparison of the reference and commercially available mouthwashes also showed that the 81% ± 3.45 viability value of ClO_2_ corresponds to the values of commercially available mouthwash products.) For apoptosis results, the mean of the reference substances tested (61.2% ± 4.44) is below the value of commercially available mouthwashes (108.7% ± 9.01). In the case of Gum Paroex, which has a strong apoptotic effect (148% ± 8.99), the proapoptotic nature of vitamin E analogs seems to be responsible among its ingredients (see Table 1 and Table 4) [51]. Nevertheless, the weak viability-reducing effect of Gum Paroex seems to balance its apoptotic character on HGEPp cells, especially when compared to the other reference substances. (Of course, the authors of the present study cannot rule out other mechanisms that were not analyzed in this study but may cause cell death, such as necrosis, necroptosis, anoikis etc.)

Taking into account the combined aspects of beneficial therapeutic effects and patient safety, (based on the present study), the reference compounds/mouthwashes have (i) weak and short-term cytotoxic effects, (ii) slight proliferation-enhancing character, and (iii) small adequate apoptosis-inducing effect on human cells.

In our experiments, we observed cell morphology changes induced by reference substances as well as mouthwashes containing reference substances. As shown in the “Result” section, “Area” and “Perimeter” values proved to be sensitive variables for cell morphology. However, of the reference compounds, ClO_2_ caused the smallest change, and mouthwashes that contained both CHX and CPC caused a significant morphological change. Data found in the literature suggest that the two reference components (CHX, CPC) also affect cell morphology by altering (i) the permeability of the cell surface membrane, (ii) cell adhesion and (iii) certain elements of the cytoskeletal system. These mechanisms mentioned above, individually or with each other, may be able to cause a reduced value for “Area” and “Perimeter.” It is clear that each of the reference compounds has a cytotoxic effect, on which their clinical application is also based. However, the duration of application can be significantly different in clinical use as well, and the maximum incubation time of 0–40 h used in our experiments far exceeds the time used in a clinical environment. Real-time impedance measurements show that high concentrations are able to exert cytotoxic effects manifested significantly earlier. Nevertheless, the ascending trends of real-time curves (e.g., Fig. 2a, d) indicate that these substances presumably result in selected subpopulations of HGEPp cells. Based on the results, ClO_2_ seems to be the most favorable considering its Safety Index value and morphometric data as well. The cell physiological effects of additional components found in mouthwashes were not studied in the framework of the present study. However, as shown in Table 4, these substances have significant cell physiological effects on human cells. Of these, the cytotoxic effects are prominent, but they may also affect proliferation, too. In mouthwashes, individual combinations of these substances can contribute significantly to viability changes of gingival epithelial cells (and presumably other types of human cells).

## Conclusions

The principal aim of the present study was to investigate the effect of a few very commonly used mouthwashes (8) and their main active components (4) on eukaryotic gingival epithelial progenitor cells (HGEPp). The oral epithelium being a squamous epithelium (providing most of the protecting barrier function), recent literature [51] shows that the barrier function still develops in HGEPp cultures, which made these cells ideal models for our experiments. A complex panel of three cell physiology assays (real-time impedimetry, computer-based morphometry, and apoptosis test) proved to be suitable to characterize the cytotoxic or viability modulator behavior of each investigated compound. With this panel of methods, it was shown that some substances are able to exert cytotoxic and apoptotic effects on human cells even at concentrations significantly lower than those in everyday use and in clinical practice. To achieve these effects, significantly longer incubation times (24–48 h) were required compared to the 1-–10-min incubation times recommended for everyday use. The potential occurrence of the observed effects poses a real risk to the unsupervised user. As in the case of unintended use, the low concentrations of the residual components of the mouthwashes in the oral cavity can reach the exposure time described in our measurements and thus can exert harmful effects on human gingival epithelium. The most significant epithelial cell viability influencing effects, based on the study of representative compounds used in dentistry, were ClO_2_ and Gum Paroex-induced apoptosis while H_2_O_2_, CHX and Perio Aid 0.12% had strong direct cytotoxic effects. The aim of the present study was not to investigate the cell physiological effects of the additional components of mouthwashes. However, numerous literature data suggest that these compounds (e.g., allantoin, ethyl alcohol (27%), NaF) may have a significant cell viability influencing effect on human cells (e.g., epithelial cells). The safe applicability of materials introduced into the human body is well characterized by the Safety Index (SI), calculated from the non-toxic values of IC50 and maximal non-toxic concentration [50]. Based on the SI and morphometric data, hyper pure ClO_2_ proved to be the most favorable among the tested materials.

## Supplementary Information

Below is the link to the electronic supplementary material.

Supplementary file1 (DOCX 2186 kb)

## Data Availability

The datasets used and/or analyzed during the current study are available from the corresponding author on reasonable request.

## Abbreviations

CHX: Chlorhexidine
CI: Cell Index
ClO_2_: chlorine dioxide
CPC: Cetylpyridinium chloride
EDTA: Ethylenediaminetetraacetic acid
GPCR: G-protein-coupled receptor
HGEPp: Human gingival epithelial progenitor cell pooled
H_2_O_2_: hydrogen peroxide
IC50: Half-maximal inhibitory concentration
PBS: Phosphate-buffered saline
PE: Phycoerythrin
RANKL: Receptor activator of nuclear factor kappa-B ligand
SI: Safety Index
