# Supplementary material for: The effects of mouthwashes in human gingiva epithelial progenitor (HGEPp) cells
Source: Clin Oral Investig. 2022 Mar 7;26(6):4559–74. doi: 10.1007/s00784-022-04422-z (PMC9203393; doi:10.1007/s00784-022-04422-z)
Supplement: Supplementary file 1 — Supplementary file1 (DOCX 2186 kb) [file 784_2022_4422_MOESM1_ESM.docx]

**SUPPLEMENTARY MATERIAL**

**The effects of mouthwashes in human gingiva epithelial progenitor (HGEPp) cells**

**Zsófia Kőhidai, Angéla Takács, Eszter Lajkó, Zoltán Géczi, Orsolya Láng^#^, László Kőhidai^#^***

# Authors contributed to the project equally

Correspondence:

László Kőhidai, Department of Genetics, Cell and Immunobiology, Semmelweis University, Budapest, 1089, Hungary, Tel: +36-30-4743803; Fax: +36-1-3036968; E-mail: kohlasz2@gmail.com

**Table S1**

Precipitating concentrations of the tested substances.

|  | **Conc. [%]** | **Conc. [% v/v]** |
| --- | --- | --- |
| CHX | 2  1 |  |
| Gum Paroex |  | 0.4  0.2 |
| PerioAid 0.12% |  | 0.4  0.2 |
| PerioAid Maintenance |  | 0.4 0.2 |
| Vitis Gingival |  | 0.4 0.2 |
| Vitis Orthodontic |  | 0.4 0.2 |
| Listerine Fluoride Plus |  | 0.4 |
| Listerine Cool Mint |  | 0.4 |

(It is worth mentioning that H_2_O_2_, ClO_2_, CPC as well as Dentaid Xeros had no precipitating side effects)

**Table S2** Summary review of cytotoxic responses. elicited by reference compounds (A) and commercially available mouthwashes (B)

| A | **Cytotoxicity**  **(*****Apoptosis detected)** | | | | |
| --- | --- | --- | --- | --- | --- |
|  | **Concentration** | **Efficiency** | **Course** | | |
|  | % | Intense – I  Moderate – M  Weak – W | Full  (h) | Early  (h) | Late  (h) |
| H_2_O_2_ | 6 | **I** | **0-40** |  |  |
|  | 3 | **I** | **0-40** |  |  |
|  | 0.3 | **I** | **0-40** |  |  |
|  | 0.03 | **I** | **0-40** |  |  |
| CHX | 0.1 | M | 0-40 |  |  |
|  | 0.01 | **I** |  |  | **20-40** |
| ClO_2_* | 60 ppm | **I** | **10-40** |  |  |
|  | 6 ppm | W |  |  | 20-40 |
| CPC | 5 | **I** | **0-40** |  |  |
|  | 1 | **I** | **0-40** |  |  |
|  | 0.5 | **I** | **0-40** |  |  |
|  | 0.05 | **I** | **0-40** |  |  |
|  | 0.005 | M | 0-40 |  |  |
|  | 0.0005 | M |  |  | 15-40 |

| B | **Cytotoxicity**  **(*****Apoptosis detected)** | | | | |
| --- | --- | --- | --- | --- | --- |
|  | **Concentration** | **Efficiency** | **Course** | | |
|  | %v/v | Intense – I  Moderate – M  Weak – W | Full  (h) | Early  (h) | Late  (h) |
| Gum Paroex* | 0.02 | **I** | **0-55** | - | - |
|  | 0.002 | M | 0-55 | - | - |
| PerioAid 0.12 | 0.02 | **I** | **0-55** | - | - |
|  | 0.002 | M | 0-55 | - | - |
| PerioAid Maintenance | 0.02 | **I** | **0-55** | - | - |
|  | 0.002 | M | 0-55 | - | - |
| Vitis Gingival | 0.02 | **I** | **0-55** | - | - |
|  | 0.002 | M | 0-55 | - | - |
| Vitis Orthodontic | 0.02 | **I** | **0-55** | - | - |
|  | 0.002 | W | 0-55 | - | - |
| Dentaid Xeros | 0.2 | **I** | **0-55** | - | - |
|  | 0.02 | W | 0-55 | - | - |
| Listerine Cool Mint | 0.2 | **I** | **0-55** | - | - |
|  | 0.02 | **I** | **0-55** | - | - |
|  | 0.002 | W | - | 7-20 | - |
|  | 2E-06 | W | - | 7-20 | - |
|  | 2E-07 | M | 0-55 | - | - |
| Listerine Fluoride Plus | 0.2 | **I** | **0-55** | - | - |
|  | 0.02 | **I** | **0-55** | - | - |
|  | 0.002 | M | - | - | 20-55 |
|  | 0.0002 | M | - | - | 20-55 |
|  | 6.67E-05 | W | - | - | 20-55 |
|  | 2E-05 | W | - | - | 20-55 |
|  | 2E-06 | W | - | - | 20-55 |

**Table S3** Summary review of proliferation inducer effects elicited by mouthwashes in HGEPp cells

|  | **Proliferation inducing effect** | | | | |
| --- | --- | --- | --- | --- | --- |
|  | **Concentration** | **Efficiency** | **Course** | | |
|  | %v/v | Intense – I  Moderate – M  Weak – W | Full  (h) | Early  (h) | Late  (h) |
| PerioAid 0.12 | 0.0002 | **W-I** | **-** | **-** | **20-55** |
|  | 2E-05 | W-M | - | - | 25-55 |
|  | 2E-06 | **W-I** | **-** | **-** | **20-55** |
| PerioAid Maintenance | 6.67E-05 | W-M | - | - | 20-55 |
|  | 2E-05 | W-M | - | - | 20-55 |
|  | 2E-07 | W | - | - | 20-55 |
| Vitis Gingival | 0.0002 | W | - | - | 25-55 |
| Vitis Orthodontic | 0.0002 | **W-I** | **-** | **-** | **20-55** |
|  | 2E-06 | W | - | - | 20-55 |
| Dentaid Xeros | 6.67E-05 | W | - | - | 30-55 |
|  | 2E-06 | W | - | - | 30-55 |
| Listerine Cool Mint | 2E-05 | W | - | - | 45-55 |
| Listerine Fluoride Plus | 2E-07 | **W-I** | **-** | **-** | **35-55** |

**Figure S1** Morphological changes in HGEPp cells in the case of CHX, H_2_O_2_, ClO_2_ and CPC treatments.

Morphometric changes were followed with Zeiss Axiovert A1 invert microscope (50x), JuLI ^TM^ FL NanoEntek and Biomorph 1.1 computer based morphometric analysis (developed by Chemotaxis reseach Group, Semmelweis University) was also applied.

**Figure S2**  Morphological changes in HGEPp cells in the case of treatments with commercially available mouthwashes.

The mouthwashes (Gum Paroex, Perio Aid 0.12%, Perio Aid Maintenance, Vitis Orthodontic) were analysed with Zeiss Axiovert A1 invert microscope (50x), JuLI ^TM^ FL NanoEntek as well as Biomorph 1.1 computer assisted program developed by Chemotaxis reseach Group, Semmelweis University.
